# The ongoing COVID-19 pandemic will create a disease surge among cancer patients

**DOI:** 10.3332/ecancer.2020.ed105

**Published:** 2020-09-02

**Authors:** Michael Meyer, Ethan Bindelglas, Michael E Kupferman, Alexander MM Eggermont

**Affiliations:** 1 Meyer Consulting, 5665 N Scottsdale Road, Suite 110 Scottsdale, AZ 85250, USA; 2 The University of Texas MD Anderson Cancer Center, 1515 Holcombe Blvd, Houston, TX 77030, USA; 3 Princess Máxima Center for Pediatric Oncology, Heidelberglaan 25, 3584 CS Utrecht, The Netherlands

**Keywords:** COVID-19, cancer, patient, policy, delayed diagnosis, surge

## Abstract

With major parts of the United States in lockdown, parts of Europe and the UK possibly going back on lockdown or expecting a second COVID-19 wave and rapidly rising rates elsewhere other than Asia, many people are forgoing regular cancer screenings and prevention services. More worrisome, some may be experiencing early signs or symptoms, yet they are not seeking evaluation, treatment or surveillance examinations. The long-term impact of this on patients, families and health care providers will be substantial. Not only will this strain sophisticated health systems in developed countries, but it will also overwhelm the health care infrastructure in developing countries.

Health-care executives, cancer center directors, oncologists and policy experts should focus now on serving this potential “third wave” of sick patients who have delayed treatment. Stopping COVID-19 is critical. However, it’s also essential to plan for the coming wave of patients who have delayed seeking care or don’t have access.

## Introduction

With the world-wide COVID-19 pandemic continuing to surge, many people have missed routine cancer screening and preventive services with their physicians. Some may be experiencing early symptoms of illness, yet still are not seeking assessments due to fear of the virus or lack of access to oncology providers. Depending on cancer type and location, a delay of six weeks in most cases is problematic but surmountable; a delay of six months may lead to stage progression and dramatic increases in death rates in cancer [1]. The number of people who die as a result of these delays could end up rivaling or exceeding deaths due to COVID-19. Adding to this potential crisis are the increased burdens being placed on health systems and medical providers which will impede their ability to provide timely and optimal care.

Much was written about the potential of COVID-19 to recede in the summer and return aggressively in the fall. The causes of any increase now appear to be more nuanced based upon geography. The reductions in new cases appear to be more due to social distancing, mask wearing, hygiene and public health measures such as lockdowns and contact tracing. Even in those places where there is current respite from the ravages of the virus, there may be a major aftershock that could throw the health systems into further crisis: a flood of patients with other illnesses who are much sicker than they would be had they not delayed visits to their doctors for fear of coronavirus exposure. Health-care executives and policy experts should focus on serving this potential “third wave” of patients; otherwise, the exuberance from having “flattened the COVID-19 curve” may be short-lived. Stopping COVID-19 is critical. It’s also essential to plan for the coming wave of patients who may be suffering from untreated cancer.

In Ghana and many other Lower and Middle Income Countries (LMICs), specialized oncology services are performed in the largest urban centers. Restrictions on movement due to COVID-19 to major cities where cancer hospitals are located is likely to negatively impact the health and wellbeing of cancer patients. Furthermore, with limited skilled staff, any COVID-19 exposure has the potential to collapse the oncology service. COVID-19 has brought increased challenges to LMICs in areas of access, staffing, psychosocial needs, diagnosis, and treatment [2].

## Challenges and early solutions during the pandemic

Cancer screenings, diagnosis, and treatment are being delayed. While certain tumors are indolent and slow growing, others are aggressive and call for early treatment. A recent UK modeling study concludes, “Our estimates suggest that, for many cancers, delays to treatment of 2–6 months will lead to a substantial proportion of patients with early- stage tumors progressing from having curable to incurable disease.” [3] Additionally, a related UK based group has calculated that a 3 to 6 month delay in cancer surgery will have an average loss of life years gained of 0.97/2.19 LYG per patient [4]. While delays may be inevitable, optimizing treatment through use of available evidence will minimize adverse outcomes. Also of major concern is the impact of the pandemic on the cancer research enterprise, with the near-total cessation of clinical trial activity during the early phase of the pandemic in the US. Estimates suggest that patient enrollment in oncology trials has deteriorated by 10% each month, since the pandemic began [5, 6]. What has emerged, however, are a number of improvements to the logistics and operations of many research programs, such as the implementation of streamlined trial activation processes, remote investigational drug delivery, remote patient consenting, and virtual patient monitoring, among others [7].

Oncologists around the world are taking steps to address this challenge. In New York, NYU has created a virtual clinic for people to call with suspicious symptoms [8]. In Hong Kong, Head and Neck surgeons have segmented tumors into 3 tiers based on their tendency to progress. Based on their experience during the SARS outbreak in 2003, they have established goals for treatment completion in each tier and have established intra city cooperation among surgeons to expedite surgery and treatment [9]. A multi institutional group of urologic oncologists from Europe and the US has evaluated evidence for worsening outcomes from delays in various urologic malignancies and provides evidence-based guidance for timing of treatment of patients with specific urologic malignancies [10]. At the University of Texas MD Anderson Cancer Center (MDACC) in Houston, disease teams have developed treatment guidelines to prioritize safe and optimal oncologic therapeutic decision-making for vulnerable populations [11–14]. By leveraging its national cancer network, MDACC has facilitated the care of its patients with collaborative health systems within its network. This has allowed patients to continue with therapeutic interventions, surveillance care and maintenance on clinical trials, thus minimizing high-risk travel during the early phase and peak of the pandemic.

### We must take steps now to avoid a substantial increase in deaths from other illnesses

Here are our recommendations for what government and industry should do to prepare for the next eighteen months and after:

- Initiate public awareness campaigns to promote the message that people should not delay care for suspicious symptoms and resume routine cancer screenings.
- Reassure patients who may be avoiding care due to fears of contracting COVID-19 in health care settings that expanded testing and other protective measures make it possible to be treated in a safe environment.
- Offer additional financial and technical support to providers, allowing them to increase capacity, design and accelerate telemedicine and patient triage programs.
- Encourage partnerships among providers, insurers, pharmaceutical companies and employers to improve coordination and quality of care. Discussions already occurring among these shareholders should be accelerated [15].
- Reduce infusion-based treatment in favor of oral therapy where possible [16].
- Restart clinical trials that have been halted or deferred due to shifts in funding.
- Encourage technological and operational enhancements for the conduct of oncology clinical trials to enhance activation and enrollment for novel investigational therapeutics.
- Augment the implementation of artificial intelligence platforms to identify patients at highest risk due to delayed treatment.
- Equilibrate reimbursement rates for a physician’s services the same regardless of where a patient receives treatment—whether at a doctor’s office or over video platforms—allowing patients to make care-based decisions rather than financially-based decisions.
- Extend health care coverage for anyone who has lost private coverage as a result of layoffs, and speed enrollment onto public insurance products for a limited time so they do not delay seeking treatment.
- Provide community-based support to promote mental health and early identification of worsening symptoms in chronic conditions.

## Conclusion

COVID-19 is having a profound impact on healthcare systems worldwide and has the potential to negatively impact patients who have existing or newly diagnosed cancers. Time delays in treatment may result in stage migration and/or a more complicated course of treatment. There are mitigation strategies we believe that will help both clinicians and patients. These include: increased cooperation and multidisciplinary care, regulatory changes, public outreach, additional support for developing countries health care infrastructure, and psycho-social support, among other things. As cited earlier, we are seeing the rapid development of evidence-based protocols to guide and prioritize cases in various areas of cancer treatment.

Continued creativity, diligence, and flexibility will be needed to ensure patients do not suffer from the consequential effects of COVID-19.

## Conflicts of interest

Mr. Meyer is a consultant to various academic/health systems, health plans and Pfizer in the US

Dr. Bindelglas has been a consultant to Pfizer, Horizon Blue Cross, and Summit Medical Group.

Dr. Kupferman has no conflicts of interest to report.

Dr. Eggermont has no conflicts of interest to report.

## Funding statement

No funding was received for this work.

## References

[1] Sud A, Torr B, Jones ME (2020). Effect of delays in the 2-week-wait cancer referral pathway during the COVID-19 pandemic on cancer survival in the UK: a modelling study. Lancet Oncol.

[2] Kugbey N, Ohene-Oti N, Vanderpuye V (2020). COVID-19 and its ramifications for cancer patients in low-resource settings: Ghana as a case study. Ecancermedicalscience.

[3] Sud A, Torr B, Jones ME (2020). Effect of delays in the 2-week-wait cancer referral pathway during the COVID-19 pandemic on cancer survival in the UK: a modelling study. Lancet Oncol.

[4] Sud A, Jones M, Broggio J (2020). Collateral damage: the impact on outcomes from cancer surgery of the COVID-19 pandemic. Ann Oncol.

[5] Unger JM, Blanke CD, LeBlanc M (2020). Association of the coronavirus disease 2019 (COVID-19) outbreak with enrollment in cancer clinical trials. JAMA Netw Open.

[6] Cahan E (2020). Clinical trials rebound after COVID-19 crash, but can enrollment gains continue?. Science.

[7] Waterhouse D, Harvey RD, Hurley P (2020). Early impact of COVID-19 on the conduct of oncology clinical trials and long-term opportunities for transformation: findings from an American Society of Clinical Oncology Survey. JCO Oncol Pract.

[8] Neel B (2020). Personal Communication.

[9] Yang Y, Shen C, Hu C (2020). Effect of COVID-19 epidemic on delay of diagnosis and treatment path for patients with nasopharyngeal carcinoma. Cancer Manag Res.

[10] Wallis CJD, Novara G, Marandino L (2020). Risks from deferring treatment for genitourinary cancers: a collaborative review to aid triage and management during the covid-19 pandemic. Eur Urol.

[11] Paul S, Rausch CR, Jain N (2020). Treating Leukemia in the Time of COVID-19. Acta Haematol.

[12] Ramirez PT, Chiva L, Eriksson AGZ (2020). COVID-19 global pandemic: options for management of gynecologic cancers. Int J Gynecol Cancer.

[13] Maniakas A, Jozaghi Y, Zafereo ME (2020). Head and neck surgical oncology in the time of a pandemic: Subsite‐specific triage guidelines during the COVID‐19 pandemic. Head Neck.

[14] Jozaghi Y, Zafereo ME, Perrier ND (2020). Endocrine surgery in the coronavirus disease 2019 pandemic: surgical triage guidelines. Head Neck.

[15] Zafar SY, Sanoff HK (2020). Changing the course of cancer care with greater precision. JAMA Oncol.

[16] Jonna S, Reuss JE, Kim C (2020). Oral chemotherapy for treatment of lung cancer. Front Oncol.

